# On the Establishment of a Provincial Jennerian Circle

**Published:** 1805-09-01

**Authors:** T. Harrison

**Affiliations:** Kirby-moor-side, Yorkshire


### *To the Editors of the Medical and Physical Journal.*

GENTLEMEN,

PRESUMING on your indulgence and the honor you have done me by the insertion of my last, I now hasten to offer my sentiments respecting the propriety of establishing what I then proposed, a *Provincial Jennerian Circle.* Some of your correspondents resident in large populous manufacturing towns or cities may possibly differ with me as to the necessity of such an establishment, and be ready to observe, that a communication with the metropolis, per post, is open to every one, and that through this medium the faculty may, on the shortest notice, be expeditiously supplied. That these are blessings of considerable magnitude I am ready to allow, nay more, that I have repeatedly availed myself thereof; but let me not conceal what others, situated in similar remote situations, must be ready to acknowledge, viz. that disappointment from hidden causes already have, and may again hereafter occur; such, for instance, as the unavoidable exposure to the extreme variations of temperature from heat to cold; from the seal of office injudiciously applied; from mechanical injury done to the infecting medium, and from other equally obvious but unavoidable contingencies. It is not however to be understood, that the number of Public Institutes which are at present so happily established in various parts of the metropolis, ought to be lessened; on the contrary, it is much to be wished that those I am now alluding to, should, if possible, be considered as collateral branches issuing from, and connected with the *Royal Jennerian Society in London,* subject to similar rules and regulations, as professing to have one common object in view, the general general diffusion of the *vaccine practice* and the total extermination of the *small-pox*.

In many small market towns similar to this, where population is extremely limited, it will not be in the power of any individual practitioner to keep a regular supply of *vaccine virus* for any considerable time together; but were five or six towns of this description adjoining each other to unite, so as to compose from their locality a kind of circle, it would be an easy matter for the respective individuals to keep up the vaccine inoculation for a limited time, and during such period, to furnish their professional brethren of the circle with a supply of recent vaccine fluid. The next member on the circle should in rotation likewise (for a period to be adjusted) keep up the vaccination, so as to complete the circle once annually. The medical few composing such a society, should have a president and secretary, to be appointed at their annual meeting; a collective list of patients vaccinated during the year should be published; vaccine topics only should be discussed, and such cases registered as the members might deem worthy of record. It would moreover be an easy matter under such an arrangment, when thought proper, for the practitioner undertaking to keep up the virus, pro tempore, to accommodate his right and left hand brother of the circle with a supply of cow-pox fluid in its most desirable state, viz. in that of its own *encircling cell* or *vesicle*, by meeting half way those vaccinated, and those wanting to partake of vaccination. These circles should be named from the district, wapontake, riding, or division, where placed, with the additional adjective *Jennerian*, both as expressive of their nature, and as a proper compliment due to the celebrated Discoverer. They should be under the controul of such rules and restrictions as the peculiar circumstances of the society might call for; and if the members composing such circles were to publish unitedly their intention of adopting the practice in their own provincial papers, there is reason to believe, that such a measure would greatly contribute to increase the public confidence. Some of your readers may smile at my proposal, and think it Utopian; I have conversed with a few of my neighbouring brethren however, who think otherwise; and whilst I am addressing and inviting them here to unite with me in carrying these proposals into effect, should the plan in your estimation seem calculated to meet a more public extension, you will do well to insert it.

 This

This address might possibly be thought to have been subjected with more propriety to Dr. Jenner individually; I cannot however allow myself to doubt his readiness to acquiesce with any proposal that may contribute to disseminate a practice, the utility of which is incontrovertibly established, and which, from his own public example, he has uniformly promulgated and supported for a series of years with the happiest success. Before I conclude this letter, excuse me for observing, that unless the celebrated Discoverer had committed himself egregiously, in his nomenclature on vaccination, there can be no reason for endeavouring, by an assemblage of new coined terms, to supersede him in his technicals. *Vacciola, vaccæ vara, bounosus,* &c. are fanciful, unnecessary, and less descriptive of the subject than *variola vaccina*. *Vaccine matter*, a term unappropriate, and apt to mislead, since it is generally known and allowed, that a vesicle possessing its true characteristic, is distinguished by a clear transparent colourless fluid, and that in proportion as it acquires a degree of thickness and opacity, approximating to pus or matter, it should be avoided. *Vaccine fluid* is therefore more consonant and expressive. As well might any one indulge himself in new phrases as his fancy might dictate, and the term *equi-vaccine*, and its attendant compounds, might be rung upon in successive changes, ad infinitum.

Herschel has named the star he discovered *Georgium Sidus*; will any astronomer dispute the title, or fail to recognize the object discovered by the term employed?

Linnæus has given numerous titles to plants, founded on his sexual system, which a scientific botanist will scarcely depart from; and shall our Jenner have his *variolæ vaccinæ* trampled upon, to accommodate the visionary whims or conceptions of any one, merely, as it has been suggested, *Euphoniæ Gratia*. There is something sacred in the terms, as employed by the Original Discoverer, which even if barely intelligible, should not be exchanged; but when these are strictly proper, and explanatory, why should we quarrel with them, unless it be to set up an ostentatious and unmeaning display of our own classical acquirements.

I am, &c.

T. HARRISON.

*Kirby-moor-side, Yorkshire, July* 16, 1805.

*Copy*

